# Precision vertical drawing of diameter-gradient microfibers: cascaded geometries for tailored nonlinearity

**DOI:** 10.1007/s12200-025-00160-8

**Published:** 2025-08-04

**Authors:** Hao Chi, Xinying He, Dezhou Lu, Shuoyang Wang, Jiahui Wu, Mengyang Jin, Xueliang Li, Zhuning Wang, Yaoguang Ma

**Affiliations:** State Key Laboratory for Extreme Photonics and Instrumentation, College of Optical Science and Engineering, Intelligent Optics and Photonics Research Center, ZJU–Hangzhou Global Scientific and Technological Innovation Center, International Research Center for Advanced Photonics, Zhejiang University, Hangzhou, 310027 China

**Keywords:** Micro/nanofiber, Fiber tapering, Supercontinuum generation, Cascaded micro/nanofiber

## Abstract

As nonlinearity is highly correlated with their geometric dimensions, precise fabrication of optical micro/nanofibers (MNFs) has been a longstanding pursuit. Existing MNFs fabrication systems typically adopt horizontal structures, which inherently introduce inaccuracy stem from asymmetry between fiber axis/geometry and chaotic environment due to high temperature airflow, vibration, etc., leading to deviations from the expected fiber morphology, especially for complex-structured MNFs. Here, we propose and manufacture a MNFs fabrication systems, effectively reducing fiber shape deviations during the fabrication process, enabling the fabrication of precise MNFs. To demonstrate the capability of our system in manufacturing precise structure MNFs, we design and fabricate diameter-gradient microfibers with four cascaded structures over a length of approximately 120 mm and a minimum diameter of about 1 μm for on-demand nonlinearity to generate supercontinuum spectrum. Eventually, we obtain supercontinuum spectrum covering 1463–1741 nm at the − 10 dB level with an efficiency of 264.62 $\text{nm}/\text{kW}$, exhibiting good flatness and enabling efficient spectral broadening.

**Graphical Abstract:**

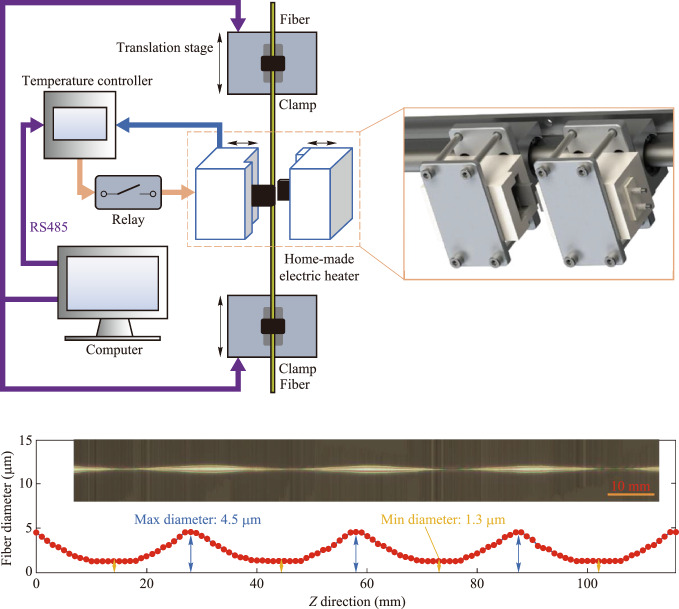

**Supplementary Information:**

The online version contains supplementary material available at 10.1007/s12200-025-00160-8.

## Introduction

Optical micro/nano fibers (MNFs), with diameters comparable to the wavelength of transmitted light, exhibit unique optical properties. These properties make MNFs a key research topic in micro-nano optics, with applications spanning sensing [1], near-field coupling [2], nonlinear optics [3], and many other areas [4]. Their distinctive geometric structure endows MNFs with various intriguing physical properties [5], including a substantial evanescent field proportion, strong optical field confinement, low optical loss, and diameter-dependent dispersion and nonlinear coefficients. Thus, the precise control of an MNF’s morphology directly influences its physical properties. For example, customizing end-face output patterns by altering fiber morphology is applied in generating optical probes [6] and micro-spectrometers [7].

For silica MNFs, high-temperature drawing of a commercial fiber using flame heating [8, 9], electric heating [10–12], and laser heating [13] is the predominant manufacturing technique because MNFs produced in this way exhibit unparalleled geometric uniformity and surface smoothness, which are crucial for low-loss optical waveguides [14, 15]. Heating softens the optical fiber while raising the air temperature, causing upward thermal airflows. Due to the structural strength of the fiber, fibers with simple geometries are not significantly affected by these airflows. Thus, all reported works have adopted horizontal methods for fiber processing. However, when a fiber undergoes multiple processing steps to create complex structures, upward thermal airflows can induce asymmetrical bending, causing deviations from the ideal shape. Although some approaches [16] employ miniature ceramic tubes to create a highly uniform heating zone and effectively suppress thermal airflow, our experience shows that the extremely narrow drawing space leads to a lower fiber fabrication success rate and limits the possibility of simultaneously drawing multiple fibers [17]. Repeated processing of MNFs can generate complex geometrical shapes and dimensions, effectively tuning their dispersion properties and nonlinear coefficients [5]. Thus, precise control of MNF processing techniques is essential for applications involving nonlinearity, such as supercontinuum generation [18, 19], four-wave mixing (FWM) [20], ultrafast lasers [21–23], and quantum optics [24, 25].

The molten core approach [26], a widely used method for manufacturing standard fibers, naturally proceeds vertically [27, 28], and minimizes stress perpendicular to the fiber drawing direction when shaping the melted silica preform. The molten core approach enables the drawing of fibers with uniform cross-sections over kilometer-scale lengths. However, optical fiber towers are typically tens of meters high and costly to build and operate.

Here, we demonstrate a table-top vertical MNFs drawing system, different from the horizontal drawing structure of traditional laboratory tapering systems, inspired by the vertical structure commonly used in industrial fiber drawing systems. Because the direction of thermal airflow is parallel to that of the fiber, this setup effectively reduces the morphological deviation during the drawing process, allowing for the production of precisely shaped MNFs. We characterized the fibers drawn using our system. The resulting MNFs closely match the designed specifications, featuring four cascaded structures over a length of approximately 120 mm and a minimum diameter of about 1 μm.

To validate our system’s ability to produce structured MNFs, we designed and fabricated diameter-gradient MNFs with cascaded geometries for tailored nonlinearity to generate supercontinuum. Simulations evaluated how structural parameters, such as taper slope and cascade number, influence supercontinuum generation. Using a genetic algorithm, we optimized these parameters under defined pumping conditions to achieve a flat supercontinuum. Based on the simulations, we fabricated cascaded MNFs with our vertical drawing system and measured their dispersion. By coupling 1550 nm pulses with 1050 W peak power and 0.12 ps width, we achieved a flat supercontinuum spanning 1463–1741 nm at − 10 dB with an efficiency of 264.62 nm/kW. Experimental studies confirmed the importance of micro-nano morphology control, while our system produced more compact, efficient MNFs compared to fused cascaded fibers [29, 30].

## Experimental section

### Vertical MNFs drawing system

**Fig. 1:**
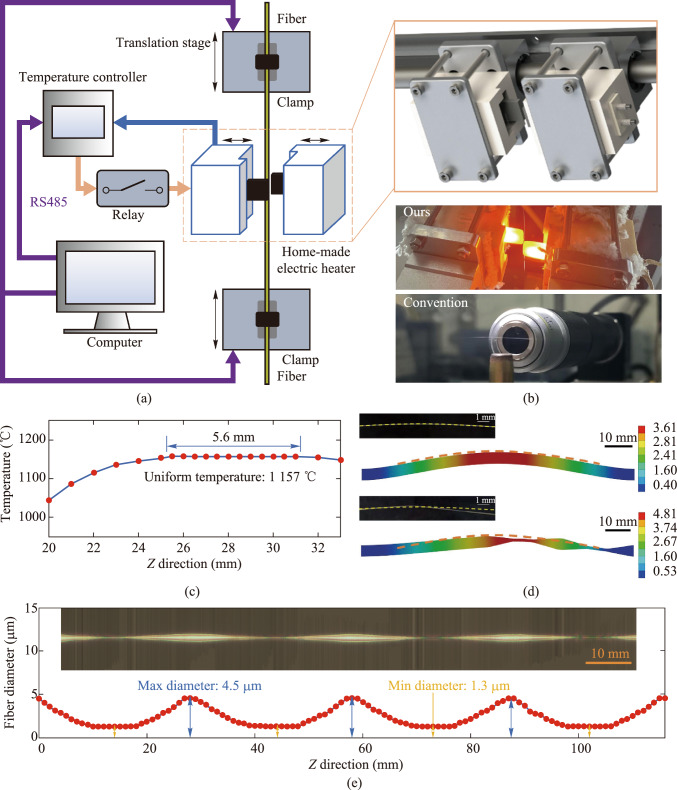
Vertical MNFs drawing system. **a** Schematic diagram of the vertical MNFs drawing system. The system consists of a home-made electric heater (the two sliders on the left, closed when heated), two translation stages (the fiber is fixed at both ends by two fiber clamps), temperature and motion control components (including temperature controller, relay, RS485, etc.), and a computer. The orange box on the right shows the 3D detail of the home-made heater. **b** Comparison of fiber morphology during drawing. The top image shows photos of fiber drawing in our system heating process, and the bottom image shows photos of fiber drawing in the traditional system heating process. **c** Temperature distribution in the *Z* direction of the heating zone. The uniform temperature is 1157 °C, the lateral temperature variation within 4 mm is less than 0.1%. **d** Real and simulated images of conventional and asymmetric-structure fibers affected by thermal airflow. The unit of the color bar on the right side of the image is mm. The dashed line indicates the bending profile of the conventional fiber. **e** The physical image and diameter measurement chart of the cascaded micro-nano fiber. In the image, the lateral and axial scales differ, with the lateral scale being about 350 times that of the axial scale. The thickest part is approximately 4.5 μm, and the thinnest part is about 1.3 μm

The schematic diagram of the vertical MNFs drawing system is shown in Fig. 1a, which mainly consists of a home-made electric heater, two translation stages, temperature and motion control components. The heater is made by bonding home-made ceramics and silicon nitride heating elements with high-temperature structural adhesive, composed of two identical parts. The heater in its open state is shown in the orange box at the top right of Fig. 1a. During heating, the two parts merge to form a single unit, creating a cavity of approximately 4 mm × 4 mm × 40 mm through which the fiber passes. The temperature inside the cavity is measured in real time by a type-B thermocouple installed in the cavity and a temperature controller (Anthone LU-926U). The typical lifetime of the heaters is approximately 1000 h under standard operating conditions, with a unit cost below 21 USD. The average power of the silicon nitride heating element is controlled by a relay (Delixi CDG1-1DA) according to the target temperature set by the computer, enabling stable temperature control. The RS485 bus is used for communication between the temperature controller and the computer. The fiber is fixed on a fiber clamp (Fujikura FH-60-250) and pulled by two high-precision translation stages (Yaskawa SGLFM-35540AC) controlled by a motion control card (Leadshine DMC3000) and a servo (Yaskawa SGDV-R90A05A), according to the preset drawing method.

The choice of fiber drawing method significantly influences the resulting fiber geometry [31]. The control program, written in C++, supports multiple drawing methods, such as the constant hot-zone method, linear hot-zone variation method, and scanning simulated constant hot-zone method, while simultaneously managing both the displacement stage and the heater.

As the temperature rises, upward thermal airflow causes the fiber in the heating zone to soften, making it prone to deformation, which affects the drawn fiber’s shape and may even lead to breakage, as shown in the lower part of Fig. 1b. To investigate the effects of thermal airflow on fibers with complex structures fabricated through multiple drawing steps, we conducted structural and stress analysis using ANSYS Workbench (Ansys Inc., Canonsburg, PA, USA). Using the measured temperature distribution (Fig. 1c), we estimated the stress distribution and analyzed the effects of thermal airflow-induced stress on both conventional and cascaded fibers. Figure 1d illustrates the structural changes occurring after 0.2 s. The upper part of the figure shows the structural changes in a conventional fiber, which bends symmetrically upward under the influence of thermal airflow. Since the fabrication requires multiple sequential drawing steps to produce four cascaded structures, we simulated an intermediate stage with two cascaded structures. The structural deformation of the fiber due to thermal airflow under asymmetric lateral conditions is shown in the lower part of Fig. 1d. Compared to conventional fibers, those with two cascaded structures exhibit greater structural deformation under thermal airflow. Due to their asymmetric horizontal structure, they experience a more complex stress distribution, resulting in not only upward bending similar to conventional fibers but also asymmetry in the horizontal direction. Since the fiber is drawn at a high temperature, any unintended deformation occurring at this stage becomes fixed during the final annealing process, causing deviations from the intended structural model.

To address this issue, we implemented two strategies. First, we enclosed our home-made heating element in a ceramic casing, which effectively insulates and reduces air convection within the fiber heating zone. Second, we designed a vertical drawing configuration, aligning the direction of thermal convection parallel to that of the fiber, as shown in the upper part of Fig. 1b, further minimizing disturbances caused by heated air on the fiber. When utilizing our system for fiber drawing, the fiber can maintain stable morphology, a critical factor for the stable and precise drawing of fibers with the desired morphology.

By measuring the temperature distribution and characterizing the fiber, we can determine the heating zone length, allowing us to draw the desired fiber shape based on the theoretical model [31]. To fabricate a fiber with four cascaded structures, we employed both the linear hot-zone variation drawing method and the constant hot-zone drawing method [31] in five steps. First, using the linear hot-zone variation drawing method, a standard fiber with a diameter of 125 μm was drawn into a fiber approximately 45 mm in length and 4.5 μm in diameter. Subsequently, using the constant hot-zone drawing method, four tapered regions, each measuring 8 mm in length and 1.3 μm in diameter, were sequentially drawn in four steps.

Finally, we present the physical image and diameter measurement chart of an MNF with four cascaded structures in Fig. 1e. This image is composed of 850 microscope images taken with a 20× objective lens, stitched together. The resulting MNF has a maximum diameter of 4.5 μm and a minimum diameter of 1.3 μm.

### Dispersion measurement of MNFs

**Fig. 2:**
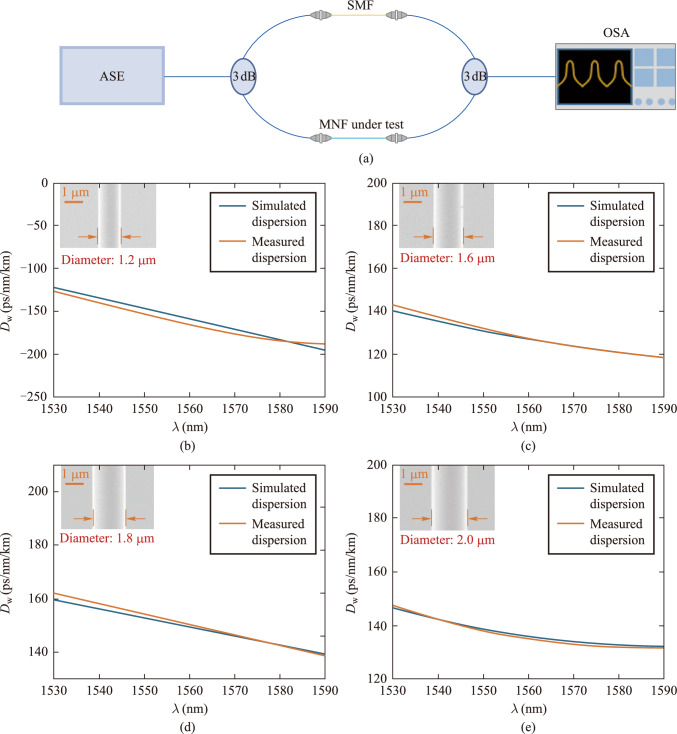
Dispersion measurement of MNFs. **a** Schematic diagram of the measurement setup based on white light interferometry. ASE: Amplified spontaneous emission; 3 dB: 3 dB coupler; SMF: single-mode fiber; OSA: Optical spectrum analyzer. **b–e** Dispersion measurement and simulation results of fibers ranging from 1.2 μm to 2 μm

Because the generation of supercontinuum and the dispersion characteristics of fibers are closely related, in order to better fit simulation and experimental results, we used white light interferometry [32, 33] to measure the dispersion parameters of the drawn MNFs. The experimental setup is shown in Fig. 2a, where free-space optical components are excluded. The intensities of the two optical paths are nearly equal, enhancing the contrast of the interference fringes. A broadband ASE (amplified spontaneous emission) light source was used, and its output was split into two paths via a 3 dB coupler. One path includes an ordinary single-mode fiber with known dispersion, while the other incorporates the MNF under test. By carefully adjusting the length of the single-mode fiber, we induce interference in both arms and extract the MNF’s dispersion characteristics through post-processing of the interference fringes. Unlike traditional white light interferometry, our setup replaces the free-space optical path in the reference arm with a single-mode fiber. Therefore, in our calculation method, we need to subtract the dispersion of the single-mode fiber to obtain the final dispersion of the MNF. We first introduced ordinary single-mode fibers of different lengths into both arms to obtain their interference spectra and dispersion distributions. Next, we introduced the MNF under test into the measurement arm and adjusted the single-mode fiber in the reference arm to optimize interference fringe contrast. Next, we extracted phase information from the interference fringes and reconstructed the phase difference according to the method proposed by Wang et al. [34], and finally obtained the group velocity dispersion of the MNF through calculation. Figure 2b–e compare experimental and simulated dispersion results for fibers ranging from 1.2 μm to 2 μm, showing deviations within acceptable limits.

### Structural design of cascaded MNF structures

To determine the structural parameters of fibers capable of producing flat and wide supercontinuum spectra, we solve the GNLSE [35] (generalized nonlinear Schrodinger equation) for numerical analysis of the parameters of cascaded MNF structures. Building upon the split-step Fourier method, we employed a fourth-order Runge–Kutta method [36] to enhance computational accuracy. To further reduce simulation time, we implemented an adaptive step-size strategy based on the conservation quantity error method (CQEM) [37].

To quantify the relative flatness of the spectrum, we defined the following index:

1 $$flatness=\frac{\sqrt{\frac{1}{n}{\sum }_{{\omega }_\text{min}}^{{\omega }_\text{max}}{\left(I\left(\omega \right)-{I}_\text{avg}\right)}^{2}}}{{I}_\text{avg}}.$$

Here, ${\omega }_\text{min}$ and ${\omega }_\text{max}$ denote the lower and upper bounds of the considered spectral range, $I\left(\omega \right)$ represents the intensity at frequency $\omega$, ${I}_\text{avg}$ stands for the average intensity over the frequency range, with smaller values indicating better flatness.

**Fig. 3:**
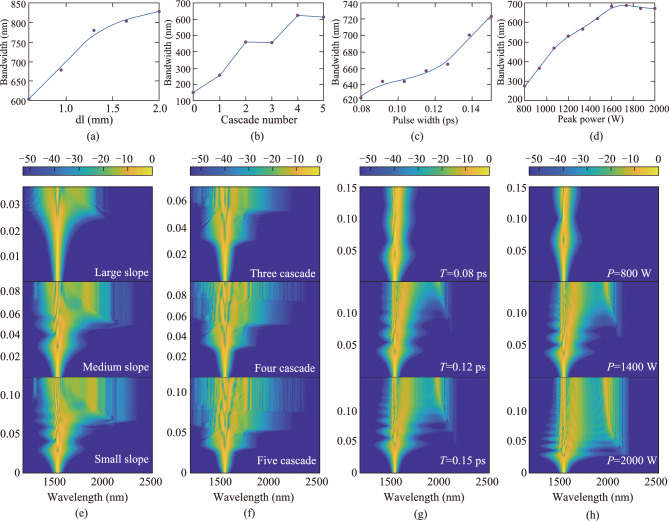
Simulation of the effects of different cascaded fiber structure parameters and input pulse parameters on spectral broadening. **a** Relationship between the taper region slope and spectral bandwidth. In the simulation, the tapered MNF is divided into small cylinders of different uniform diameters, dl represents the length of the small cylinder under the same initial taper region diameter and waist diameter, the longer the dl, the smaller the slope. Under this simulation condition, there is only one cone region, with a peak power of 2000 W and a pulse width of 0.1 ps. **b** Relationship between the number of cascades and spectral bandwidth. Under this simulation condition, all have medium slope, peak power of 2100 W, and pulse width of 0.12 ps. **c** Relationship between the incident pulse width and spectral bandwidth. Under this simulation condition, all have medium slope, with only one cone region, and a peak power of 1300 W. **d** Relationship between the peak power of the incident pulse and spectral bandwidth. Under this simulation condition, all have medium slope, with only one cone region, and a pulse width of 0.12 ps. The spectral bandwidth in **a–d** refers to the spectral width with intensity variations less than 30 dB. **e** Spectral evolution in cascaded MNFs with different cone slope. **f** Spectral evolution in cascaded MNFs with different cascading numbers. **g** Spectral evolution of incident pulses with different pulse widths. **h** Spectral evolution of incident pulses with different peak powers

We first studied the relationship between taper slopes and spectral bandwidth. As shown in Fig. 3a, decreasing the taper slope leads to spectral broadening from 604.44 nm to 826.86 nm at the 35 dB level. The spectral evolution at different slopes is shown in Fig. 3e. As the taper slope decreases from top to bottom, the corresponding spectral bandwidths are 604.44 nm, 743.22 nm, and 826.86 nm, respectively. When the taper slope is large, the second zero-dispersion wavelength shifts too rapidly along the fiber length. As a result, the red-shifted soliton may fail to overlap with the dispersion wave spectrum, preventing energy transfer beyond the second zero-dispersion wavelength. Conversely, when the taper slope is too small, the extended taper length reduces peak power, limiting further spectral broadening. Moreover, a shallow taper slope increases fabrication challenges.

Building on the optimization of taper slope, we further explored the influence of the number of cascaded structures on the generation of supercontinuum spectrum. As shown in Fig. 3b, the number of cascades increases from 0 to 5, with the spectral bandwidth gradually expanding from 151.28 nm to around 612.70 nm. Figure 3f presents the spectral evolution along the propagation distance for fiber structures comprising three, four, and five cascaded structures. With an increasing number of structures, higher-order soliton fission phenomena become progressively more prominent. In the case of the three-segment structure, only one to two soliton fission events are observed, resulting in relatively limited redshift spectral broadening. In contrast, for the four- and five-segment configurations, soliton fission occurs at earlier propagation stages. The resulting fundamental solitons subsequently undergo enhanced Raman self-frequency shifts, yielding significantly broadened redshifted spectral components. Simultaneously, once these fission-generated solitons traverse into the positive dispersion regime, phase-matching conditions with dispersive waves are satisfied, thereby facilitating the generation of distinct blue-edge spectral features. These features are indicative of Cherenkov radiation-induced dispersive wave. However, beyond a certain point, the accumulation of dispersion reduces the pulse's peak power, limiting further spectral broadening.

Since the generation of supercontinuum spectrum is the result of the comprehensive effects of various nonlinear effects, the optimization of the structure of MNFs is usually cumbersome and inevitably requires a large number of attempts. However, for MNFs with a determined diameter, the various-order dispersions and nonlinear coefficients at specific wavelengths are fixed. Therefore, some global optimization algorithms can realize the reverse design of MNF structures required for specific output spectra. We used a genetic algorithm for this reverse design optimization of the proposed cascaded fiber structure. Ultimately, we obtained fiber parameters that balance spectral width and flatness. The initial waist diameter is 4.5 μm, with a length of 42 mm, the diameter of the secondary taper waist is 1.32 μm with a length of 8 mm, and the number of cascades is 4.

Different pump wavelengths exhibit different dispersion and nonlinear characteristics when transmitted in fibers, leading to varied pulse evolution processes. Peak power and pulse width are significant factors influencing pulse evolution for a given pump wavelength. Thus, we studied how pump parameters affect the performance of supercontinuum generation in fibers. As shown in Fig. 3c, increasing the pulse width from 0.08 ps to 0.15 ps broadens the spectral bandwidth from 624.10 nm to 723.12 nm. Figure 3g shows the spectral evolution for pulse widths of 0.08 ps, 0.12 ps, and 0.15 ps, with corresponding bandwidths of 624.10 nm, 656.79 nm, and 723.12 nm respectively. For small pulse widths, the dispersion length is short, and the number of solitons is limited. Hence, the spectral width is limited. However, with increasing pulse width, the number of solitons grows. The split of higher-order solitons leads to significant spectral broadening, and there is mutual overlap between the split processes of different solitons, resulting in the appearance of fine structures in the output spectrum.

As shown in Fig. 3d, increasing the peak power from 800 to 2000 W results in an expansion of the spectral bandwidth from 276.43 nm to 671.72 nm. Figure 3h shows that for peak powers of 800 W, 1400 W, and 2000 W, the corresponding spectral bandwidths are 276.43 nm, 566.0 nm, and 671.72 nm, respectively. This is because, with other conditions unchanged, the larger the peak power, the shorter the corresponding nonlinear length, and the faster the accumulation of nonlinear phase shift, resulting in a higher soliton order at this time. Therefore, when considering specific applications, such as specific requirements for the spectral width and coherence of supercontinuum spectrum, it is often necessary to select appropriate pump power. Due to coupling efficiency and hardware limitations, we chose a pump peak power of 1050 W.

## Results and discussion

### Cascade MNFs generate supercontinuum spectrum

**Fig. 4:**
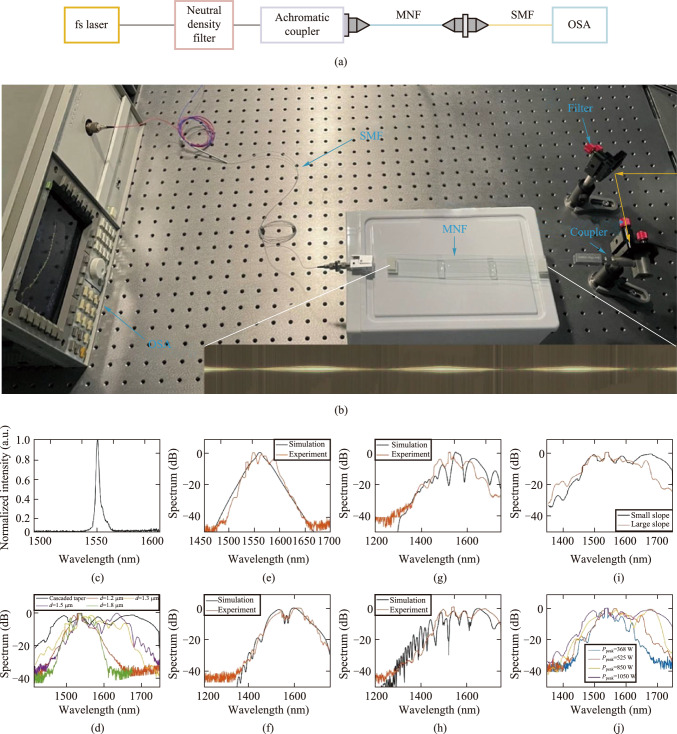
Generation and measurement of supercontinuum spectrum based on cascaded MNFs. **a** Schematic diagram of supercontinuum spectrum generation experiment. OSA: Optical spectrum analyzer; MNF: cascaded micro-nano fiber. **b** Photograph of supercontinuum spectrum generation experiment. The orange line represents the propagation of laser in free space; The inset is a physical picture of the cascaded MNF. **c** Spectrum of femtosecond laser input. **d** Comparison of broadened spectra between cascaded MNF and ordinary MNF. The black curve represents the cascaded MNF. The orange, yellow, purple, and green curves correspond to ordinary MNFs with diameters of 1.2 μm, 1.3 μm, 1.5 μm, and 1.8 μm, respectively. **e–h**, comparison of output spectra for cascaded MNFs with different numbers of cascades. **e**–**h** correspond to structures with 1–4 cascades, respectively. **i** Comparison of output spectrum of cascaded MNFs with different slopes. **j** Comparison of output spectrum of cascaded MNFs at different power levels. The blue, orange, yellow, and purple curves correspond to the output spectra with peak powers of 368 W, 525 W, 850 W, and 1050 W, respectively

We utilized the fiber drawing system mentioned earlier to fabricate the cascaded MNFs for supercontinuum generation through multi-step drawing. To validate the accuracy of numerical analysis and simulations, we further experimentally verified the role of the proposed cascaded MNF structure in generating supercontinuum spectrum. The schematic diagram of the supercontinuum spectrum measurement is shown in Fig. 4a, and the experimental setup is depicted in Fig. 4b. We utilized a femtosecond Er-fiber laser (Rainbow 1550 HP, NPI lasers) as the seed light source, with an input pulse width of approximately 120 fs and a central wavelength of 1550 nm. The laser first passed through a neutral density filter to adjust the power, then coupled into the MNF using an achromatic collimator (RC08FC-P01, Thorlabs), and finally, the output spectrum was measured using an optical spectrum analyzer (MS9710B, Anritsu).

We demonstrated the spectral broadening effect of our designed and fabricated cascaded MNFs. Figure 4c illustrates the spectrum of the input laser before passing through the MNFs. Figure 4d compares the broadened spectrum obtained after passing through the cascaded MNFs with the spectral broadening effect of ordinary optical fibers, we selected uniform MNFs with diameters of 1.2 μm, 1.5 μm, and 1.8 μm for comparison. These diameters are all within the range of the maximum and minimum diameters of the taper region obtained through our optimization. It can be seen that the spectrum broadened by the cascaded MNF is further expanded in the range of 1350 nm to 1750 nm compared to the ordinary MNF, with an output bandwidth of 277.85 nm at 10 dB level and a flatness of approximately 0.4874.

To validate the conclusions obtained in our simulations, we utilized MNFs with different numbers of cascades to generate supercontinuum spectra, as shown in Fig. 4e–h. As the number of cascades increases from 1 to 4, the experimentally obtained spectral bandwidths are 62.70 nm, 201.25 nm, 173.47 nm, and 277.85 nm at 10 dB level, respectively.

We also compared the supercontinuum spectra generated by MNFs with different slopes under the same pumping conditions. To obtain MNFs with larger slopes in the cascade structure, similarly, we first used electric heating melting and stretching to obtain a uniform diameter of 4.5 μm and a length of 42 mm MNF. Then, using the laser heating drawing system in our laboratory, we performed secondary drawing on the uniform long taper, with a heating zone length of 2 mm, a taper length of 4.9 mm, and a uniform taper waist diameter of 1.32 μm. Compared with electric heating system, laser heating system provides a shorter heating zone length, so under the same initial diameter and taper waist diameter in the lower taper zone, a shorter stretching length is required, which means a shorter taper zone, in other words, a larger slope in the taper zone. As shown in Fig. 4i, it can be seen that compared to the case of a short taper zone with a larger slope in the taper zone, the advantages of a long taper zone with a smaller slope in the taper zone in terms of spectral broadening are more obvious. The optimized cascade MNF has a bandwidth of 277.85 nm and a flatness of 0.4874, while the cascade MNF with a larger taper zone slope has a bandwidth of 215.52 nm and a flatness of 0.5835. When the slope of the taper zone is small, the length of its nonlinear interaction is longer. Additionally, the gradual taper is beneficial for better phase matching of dispersion waves and four-wave mixing, which promotes spectral broadening.

We compared the output spectrum of cascade MNFs under different pump power levels, as shown in Fig. 4j. It can be seen that as the peak pump power increases, the width of the spectrum also increases, with a more pronounced expansion in the long-wavelength region. When the pump power is higher, more solitons are injected, and the pulse delay of the split individual solitons is shorter, resulting in a wider spectrum. The experimental results show that when the pump power is 850 W, the − 10 dB spectrum width of the output is 231.14 nm, with a flatness of 0.5823, and when the pump power is 1050 W, the − 10 dB spectrum width of the output is 277.85 nm, with a flatness of 0.4874. Generally, higher pump power leads to a wider spectrum but worsens the flatness. However, when comparing the spectrum width and flatness at 850 W and 1050 W, it is evident that our optimized cascade structure not only provides a larger spectrum width but also a better flatness at 1050 W. This further highlights the advantage of our optimized cascade structure under specific pump conditions.

To quantitatively describe the significance of cascade structures in generating supercontinuum spectrum, we have defined the following parameter:

2 $$\eta =\frac{bandwidth}{P},$$

where $bandwidth$ represents the spectral range with relative changes less than 10 dB, and $P$ is the peak power of the pump, with the unit of this parameter being nm/kW.

Table 1 compares the efficiency of supercontinuum spectrum generation between previously reported results and our own. It can be seen that under the unit peak pump power, our proposed cascaded structure MNF has the highest efficiency in the process of generating supercontinuum spectrum. In addition, using cascaded structure MNF to generate supercontinuum spectra has the advantage of compact structure compared to traditional fusion methods, which is conducive to the miniaturization development of supercontinuum light sources. The research based on the cascaded structure microfiber for generating supercontinuum spectra can be applied not only in the 1550 nm band but also in other bands such as 850 nm and 1030 nm. In summary, the proposal of our cascaded structure microfiber provides a possible method for improving the efficiency of supercontinuum spectrum generation.

**Table 1:** Comparison of supercontinuum generation efficiency among different fiber structures

Fiber type	Pump peak power (kW)	Fiber length (cm)	− 10 dB level spectrum range	$\eta$ (nm/kW)	References
PCF	8	75	400–1500	137.50	[38]
PCF	3	100	500–1200	233.34	[39]
PCF	38.47	2.4	325–1200	22.75	[40]
MNF	11.14	9	400–1500	98.73	[41]
MMF	300	50	600–2300	5.67	[42]
Cascaded MNF	1.05	12	1463–1741	264.62	This work

## Conclusions

By utilizing a vertical MNFs drawing system, we designed and fabricated target-shaped cascade MNFs, resulting in the generation of relatively flat supercontinuum spectrum. On the one hand, the vertical setup can mitigate the impact of thermal airflow, allowing the creation of very intricate fiber structures. On the other hand, compared to the traditional fusion splicing method for manufacturing cascade structures, this study achieved a multi-step tapering process on the basis of the initially tapered MNFs with long waist sections. In contrast to fusion splicing [29, 30], which typically requires leaving longer tail fibers, our fiber fabrication method is more compact and reduces restrictions on fiber structure. Experimental results demonstrate the excellent capability of our vertical MNFs drawing system for precision machining of MNFs.

Through simulations and optimizations of fiber structure parameters, we determined the necessary parameters for cascade MNFs to generate a flat supercontinuum spectrum under laboratory conditions. Using our precision fiber drawing system, we fabricated target-shaped cascade MNFs. To validate the simulations, we measured their dispersion using white light interferometry. By analyzing the effects of taper slope, cascade number, and pump power, we confirmed that diameter-varied taper regions enhance supercontinuum generation, highlighting the importance of precision machining in spectral broadening. After fabricating the MNFs, we coupled 1050 W pulses with 0.12 ps width into the fibers, achieving a supercontinuum spanning 1463–1741 nm with good flatness, demonstrating higher spectral broadening efficiency than other reported fibers.

The ability to fabricate complex fiber structures with higher precision maximizes the design freedom, leading to better results. Meanwhile, the ability to stably process micro-nano fibers is also the foundation for the potential large-scale fabrication of MNFs in the future.

## Supplementary Information

Below is the link to the electronic supplementary material.

Supplementary file1 (PDF 306 KB)

## Data Availability

The data that support the findings of this study are available from the corresponding author, upon reasonable request.
