# Supplementary material for: Precision vertical drawing of diameter-gradient microfibers: cascaded geometries for tailored nonlinearity
Source: Front Optoelectron. 2025 Aug 4;18(1):16. doi: 10.1007/s12200-025-00160-8 (PMC12321704; doi:10.1007/s12200-025-00160-8)
Supplement: Supplementary file 1 — Supplementary file1 (PDF 306 KB) [file 12200_2025_160_MOESM1_ESM.pdf]

Supporting Information

Precision vertical drawing of diameter-gradient microfibers: cascaded geometries for tailored nonlinearity

Hao Chi,[1,#] Xinying He[1,#], Dezhou Lu[1], Shuoyang Wang[1], Jiahui Wu[1], Mengyang Jin[1] , Xueliang Li[1] , Zhuning Wang[1], Yaoguang Ma[1,*]

[a] State Key Laboratory for Extreme Photonics and Instrumentation, College of Optical Science and Engineering, Intelligent Optics and Photonics Research Center, ZJU–Hangzhou Global Scientific and Technological Innovation Center, International Research Center for Advanced Photonics, Zhejiang University 310027, China

[#] These authors contributed equally to the work.

[*] Corresponding author. E-mail address: *mayaoguang@zju.edu.cn*

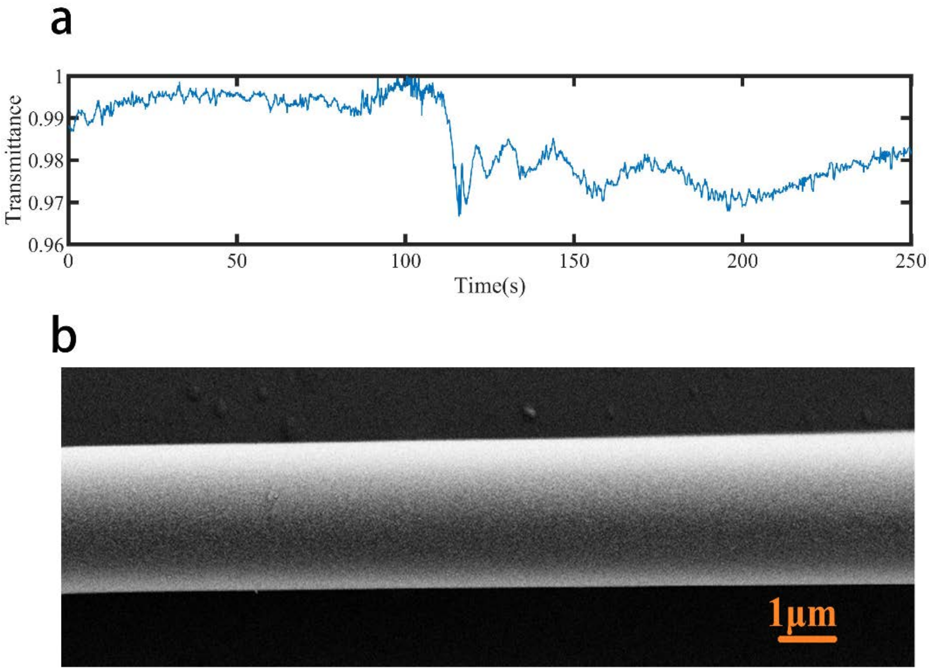

Fig. S1. a The normalized transmission as a function of drawing time. b The SEM image of the fiber after tapering.

We fabricated adiabatically tapered fibers to minimize optical loss, allowing high transmission even with a small waist diameter. For a stretched length of 55 mm, the waist diameter reached 0.96 μm. As shown in Fig. S1a, the transmission efficiency during the drawing process increases, reaching over 98% upon fiber completion. We also tested the MNF with four cascaded structures described in this work. Although the cascaded segments increased optical loss, the final transmission remained acceptable at approximately 72%. The SEM image of the fiber shown in Fig. S1b demonstrates the excellent surface smoothness of the fiber after tapering.

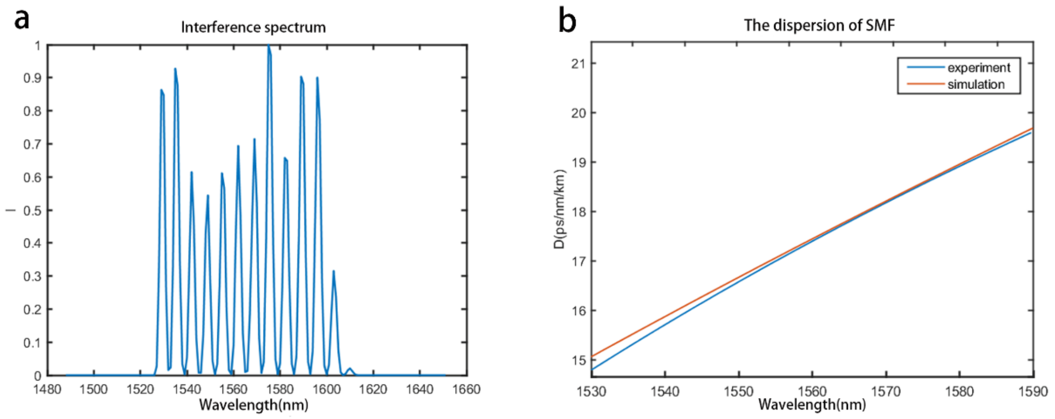

Fig. S2. Calibration of SMF dispersion. a interference spectra of SMF. b the dispersion measurement and simulation results of SMF.

Since we replaced the free-space optical path with single-mode fiber (SMF), we needed to subtract the dispersion of the SMF in our calculations to obtain the final dispersion of the MNF. To determine the dispersion of standard SMF, we introduced different lengths of SMF into the two interferometer arms. The resulting interference spectra and calculated dispersion are shown in Fig. S2a. Numerical simulations using the finite element software COMSOL Multiphysics show good agreement with the experimental results as showed in Fig. S2b.

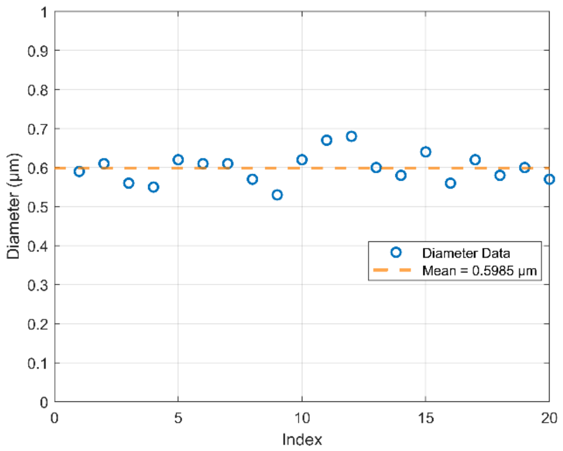

Fig. S3. Waist diameters of 20 micro/nanofibers drawn under identical parameters. The orange line represents the average diameter (0.5985 μm) with a standard deviation of 0.0382 μm.

To verify the repeatability of the tapering system, 20 micro/nanofibers with diameters below 1 μm were successfully drawn using identical parameters. Their waist diameter distribution is shown in Fig. S3. These fibers were drawn over two days with a success rate of approximately 70%, mainly limited by the fragility of the slender fibers, which are prone to touching the heater during handling. The average diameter of these 20 fibers was 0.5985 μm, with a drawing length of 53 mm and a diameter standard deviation of 0.0382 μm.
